# *FasL *and *FADD *delivery by a glioma-specific and cell cycle-dependent HSV-1 amplicon virus enhanced apoptosis in primary human brain tumors

**DOI:** 10.1186/1476-4598-9-270

**Published:** 2010-10-13

**Authors:** Ivy A Ho, Wai H Ng, Paula Y Lam

**Affiliations:** 1 Laboratory of Cancer Gene Therapy, Cellular and Molecular Research Division, Humphrey Oei Institute of Cancer Research, National Cancer Centre, 169610 Singapore; 2 Department of Neurosurgery, National Neuroscience Institute, 308433 Singapore; 3 Department of Physiology, Yong Loo Lin School of Medicine, National University of Singapore, 117597 Singapore; 4 Duke-NUS Graduate Medical School, 169547 Singapore

## Abstract

**Background:**

Glioblastoma multiforme is the most malignant cancer of the brain and is notoriously difficult to treat due to the highly proliferative and infiltrative nature of the cells. Herein, we explored the combination treatment of pre-established human glioma xenograft using multiple therapeutic genes whereby the gene expression is regulated by both cell-type and cell cycle-dependent transcriptional regulatory mechanism conferred by recombinant HSV-1 amplicon vectors.

**Results:**

We demonstrated for the first time that Ki67-positive proliferating primary human glioma cells cultured from biopsy samples were effectively induced into cell death by the dual-specific function of the pG8-*FasL *amplicon vectors. These vectors were relatively stable and exhibited minimal cytotoxicity *in vivo*. Intracranial implantation of pre-transduced glioma cells resulted in better survival outcome when compared with viral vectors inoculated one week post-implantation of tumor cells, indicating that therapeutic efficacy is dependent on the viral spread and mode of viral vectors administration. We further showed that pG8-*FasL *amplicon vectors are functional in the presence of commonly used treatment regimens for human brain cancer. In fact, the combined therapies of pG8-*FasL *and pG8-*FADD *in the presence of temozolomide significantly improved the survival of mice bearing intracranial high-grade gliomas.

**Conclusion:**

Taken together, our results showed that the glioma-specific and cell cycle-dependent HSV-1 amplicon vector is potentially useful as an adjuvant therapy to complement the current gene therapy strategy for gliomas.

## Background

Glioblastoma multiforme (GBM) accounts for more than 70 % of all primary central nervous system neoplasms in adults [1]. Despite advances in surgery, chemotherapy, and radiotherapy, the life expectancy of patients with GBM is still less than 1 year [2]. The failure of current therapeutic approaches to treat GBM is attributed to the high proliferative and infiltrative nature of these neoplasms [3]. Malignant cells are often seen surrounding the neurons and blood vessels and migrate through the white matter tracts to regions distant from the original tumor mass, thus the incidence for tumor recurrence is high. Herein, we explored the combination treatment of pre-established human glioma xenograft using multiple therapeutic genes whereby the gene expression is regulated by the status of cellular proliferation of the cancer cells.

We have previously constructed a Herpes Simplex Virus type 1 (HSV-1)-based amplicon vector in which the activation of the transgene expression is regulated by a G_0_/G_1_-specific transcriptional repressor protein termed cell cycle-dependent factor 1, CDF-1 [4]. CDF-1 repressor protein binds to the CDE/CHR regulatory region located within the *cyclin A *promoter. In quiescent cells, the transactivation of *cyclin A *promoter could not take place due to the binding of the CDF-1 repressor protein onto the *cyclin A *promoter. However, in actively proliferating cells, transcription of the luciferase reporter [4,5] or therapeutic gene [6] is activated due to the absence of the CDF-1 repressor protein. As a proof-of-concept, we have chosen the *Fas ligand *(*FasL*) and the *Fas-associated death domain *(*FADD/MORT1*) as therapeutic genes because it is important that the effects derived from these genes should not mask the cell cycle-dependent function of the amplicon viral vectors.

Fas ligand/APO-1L (CD95L) is a ~ 40 kDa type II membrane protein belonging to the tumor necrosis factor (TNF) family. Full length FasL can be further processed to release a functional soluble 26 kDa molecule known as soluble FasL [7]. Binding of FasL to its receptor Fas triggers the trimerization of the Fas receptors and initiates the recruitment of the cytoplasmic adaptor protein FADD through the interaction of the death domains [8]. Recruited FADD then interacts with procaspase-8 via the death effector domain to form the death-inducing signaling complex (DISC). The close proximity of caspase-8 zymogens facilitates their autocatalytic cleavage, which subsequently trigger the downstream effector caspases resulting in apoptosis [7,9]. Both Fas and FasL expression are absent in normal astrocytes; however, the expression of Fas, but not FasL, in astrocytomas appear to correlate with neoplasm grade [10-12]. Based on these findings, the Fas/FasL receptor system has been proposed as specific target for human brain tumor therapy. This contradicts another school of thought where the Fas/FasL receptor interaction grant the tumor cell an immune-privileged status, supported by studies demonstrating that the FasL expression in cancer cells deliver death signals to activated Fas-positive T lymphocytes [13-15]. Aside from the possible role in immune surveillance, some of the glioma cells are resistant to Fas-induced apoptosis [16,17], possibly due to low levels of Fas expression [16,18], or absence of FADD [19] or caspase 8 expression [20]. Alternatively, epigenetic aberrations can select for glioma cells that possess several resistance mechanisms to conventional therapies [21]. Interestingly, the overexpression of caspase 8 or FADD has been demonstrated to rescue the defect and rendered the cells sensitive to FasL-induced apoptosis [19,22]. Recently, inducible FADD was also shown to induce apoptosis in resistant glioma cells [18].

Since the Fas/FasL receptor pathway converges at FADD, we hypothesized that the overexpression of FADD could sensitize glioma cells to FasL-induced apoptosis. In view of the multifaceted roles of *FasL *and *FADD *in keeping the homeostasis of immune cells, these genes were inserted into a previously generated cell-cycle regulatable HSV-1 amplicon vector under a glial cell-specific *GFAP *promoter. We demonstrated that the newly generated therapeutic vectors are capable of inducing cell death in proliferating primary human glioma cells derived from patients, suggesting that these vectors are functional in a clinical scenario. Furthermore, these vectors are stable, elicit minimal immune response, and are not significantly hampered by chemotherapy or irradiation *in vivo*. More importantly, we showed that the co-expression of FasL and FADD could elicit potent anti-tumor effect, which was enhanced in the presence of temozolomide, resulting in prolonged survival of mice bearing orthotopic gliomas. Taken together, our results demonstrated that the glial-specific, cell cycle-regulatable HSV-1 amplicon viral vectors may prove useful in enhancing the efficacy of glioma treatment.

## Results

### pG8-FasL amplicon viral vectors induced apoptosis in human glioma cells in a glial cell-specific and cell cycle-dependent manner

**Figure 1:**
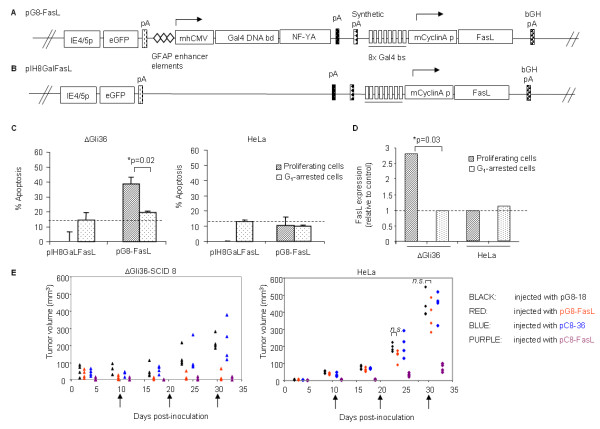
**Glioma-specific and cell cycle-regulated apoptosis mediated by pG8-*FasL***. (A) The pG8-*FasL *vector contained three-tandem repeats of the *GFAP *enhancer element upstream of the minimal *CMV *promoter to drive glial-specific activation of the transcriptional activator, *Gal4/NF-YA*. The pG8-*FasL *amplicon vector consisted of the *eGFP *gene under the control of the immediate early promoter (IE4/5p) for titering and monitoring of viral infection. (B) pIH8Gal*FasL *was generated by removal of the *luciferase *gene from pIH8Gal*Luc *(Additional File 1) and replaced with the *FasL *gene. This vector lacked the *Gal4/NF-YA *transactivator sequence, and was used as a negative control throughout this study. (C) The percentage of apoptotic cells in pG8-*FasL *and pIH8Gal*FasL*-transduced ΔGli36 and HeLa cells were analyzed 72 h post-infection. Data shown are the averages of triplicate experiments + SEM. Dotted lines indicate the background apoptosis resulted from the combined effect of lovastatin and low serum level. (D) FasL expression was determined by ELISA in both ΔGli36 and HeLa cells 72 h post-infection. (E) SCID mice harboring either ΔGli36-SCID8 or HeLa were injected with 1× 10^6 ^TU of pC8-*FasL *(purple), pC8-36 (blue), pG8-*FasL *(red), and pG8-18 (black) vectors. Arrows indicated the repeated injection time. Tumor volume was monitored at different time points.

Previously, we reported a HSV-1-based amplicon viral vector (denoted as pC8-36; Additional file 1) that mediated luciferase reporter activities in a cell cycle-dependent manner. These vectors were later demonstrated to exhibit cell cycle-dependent therapeutic efficacy when the *luciferase *reporter gene was substituted by the *FasL *gene [6]. To further investigate whether the therapeutic gene expression can be restricted not just in dividing cells, but also in glial fibrillary acidic protein (GFAP)-expressing glial cells, the ubiquitous *CMV *promoter in pC8-*FasL *amplicon vector (Additional file 1) was swapped with the astrocytes-specific *GFAP *enhancer elements and the minimal *CMV *promoter. The newly derived vector was named pG8-*FasL *(Figure 1A). In rapidly dividing GFAP-expressing cells, the glial cells-specific promoter is activated resulting in the transcription of the Gal4/NF-YA fusion protein. The fusion protein will transactivate the minimal *cyclin A *promoter through its binding to the Gal 4 DNA binding sites, thus mediating FasL protein expression in proliferating glial cells. The pIH8Gal*FasL *construct, which lacked the *Gal4/NF-YA *fusion gene, served as a control vector (Figure 1B). To confirm the cell-type specific transcription, human glioma cells ΔGli36 and non-glioma cells, HeLa, were chosen because they exhibited similar transduction efficiency when infected by the different types of HSV-1-based amplicon viral vectors (data not shown). As shown in Figure 1C, approximately 20 % higher cell death was observed in proliferating pG8-*FasL*-transduced ΔGli36 cells in comparison to the G_1_-arrested cells. On the other hand, similar levels of apoptosis was detected in ΔGli36 cells infected with either pIH8Gal*FasL *(Figure 1B) or pG8-*FasL *under the growth arrest conditions. This result suggested that the observed cell death is non-specific, and most likely due to the combined effect of low serum and lovastatin that has been shown to induce apoptosis [23]. By contrast, the pG8-*FasL *amplicon viral vectors appeared to have lost its activities in HeLa cells, presumably due to the absence of specific cellular factors that are required for the *GFAP*-containing promoter to be functional (Figure 1C, right panel). These results were in agreement with the higher level of FasL expression detected in the proliferating ΔGli36 cells (Figure 1D), providing further evidence that the FasL expression was regulated in a cell cycle-dependent and glial cell-specific manner.

To demonstrate that pG8-*FasL *could confer glial cell-specific transgene expression *in vivo*, both pG8-*FasL *and pC8-*FasL *amplicon viral vectors were used in mice bearing xenografts of two different cell type origins. The latter vector, which consisted of the CMV promoter driving the *Gal4/NF-YA *fusion gene, served as positive control because it has been shown to induce apoptosis in all proliferating cell type [6]; on the contrary, pC8-36 and pG8-18 vector (Additional file 1), which expressed the luciferase reporter protein, served as negative control for pC8-*FasL *and pG8-*FasL*, respectively. The pC8 series of vectors contained the *luciferase *reporter gene under the control of a minimal *cyclin A *promoter downstream of a ubiquitous *CMV *promoter; while the pG8 series of vectors conferred glioma-specific transgene expression, both vectors conferred cell cycle-dependent transgene expression.

Amplicon viral vectors (i.e., pG8-18, pG8-FasL, pC8-36, and pC8-FasL; 2 × 10^6 ^transduction units (TU)) were injected into immunodeficient mice harboring either HeLa-derived or ΔGli36-SCID8-derived tumors. The latter was used due to its consistency in the induction of tumor growth in immunodeficient CB-17 SCID mice. Similar to ΔGli36 cells, this derivative is also sensitive to FasL-induced apoptosis and has more aggressive tumor growth kinetics in immunodeficient mice (data not shown). Administration of viral vectors was performed at a 10-day interval based on our previous findings [6]. Our results demonstrated that pG8-*FasL *(red color) effectively suppressed tumor growth in mice bearing ΔGli36 glioma xenografts when compared with tumors injected with the pG8-18 amplicon viral vectors (black; p = 0.007; Figure 1E*left panel*). Similar trend was not observed in mice bearing HeLa xenografts. At the final measurement, the tumor volumes in pG8-*FasL-*injected ΔGli36 tumors ranged between non-detectable to 69.06 mm^3^, whereas the tumor volume in pG8-*FasL*-injected HeLa tumors ranged between 282.52 mm^3 ^and 500.09 mm^3^. In both cell types, tumors injected with pC8-*FasL *(purple) were significantly smaller in size when compared to tumors injected with pC8-36 (blue). This result demonstrated that the expression of the *FasL *gene under the ubiquitous *CMV *promoter induced a uniform suppression in the growth of both cell types. Thus, the cell death mediated by pG8-*FasL *is restricted to proliferating tumor cells of glial origin.

### Therapeutic efficacy of pG8-FasL amplicon vector on primary human glioma cells

**Figure 2:**
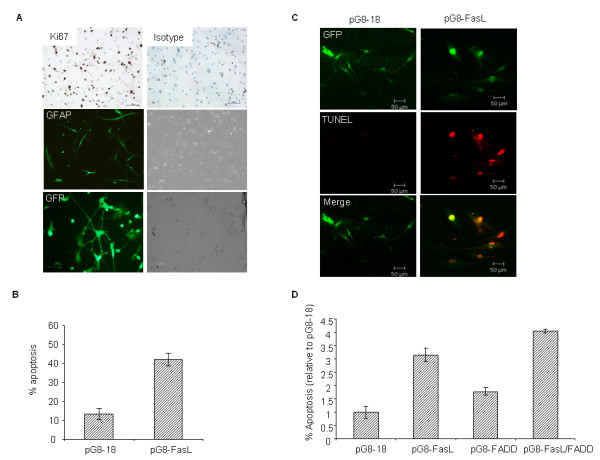
**Therapeutic efficacy of pG8-*FasL *in primary GBM**. (A) Images showed the immunostaining against Ki67 (*top *panel) and GFAP (*middle panel*) in primary human glioma cells *Bottom panel*, primary human glioma cells were infected with MOI of 0.8 of pG8-18. Images showed the percentage of eGFP+ cells. Original magnification ×100 was shown. (B) The percentage of apoptotic cells in pG8-*FasL *and pG8-18-transduced primary glioma cells. Data shown are the averages of triplicate experiments + SEM. (C) Fluorescence images showed eGFP+ and TUNEL+ cells on pG8-*FasL *and pG8-18-transduced primary glioma cells 72 h after infection. Transduction efficiency of primary human glioma cells as visualized by eGFP expression. Original magnification ×100 was shown. Images were captured using Nikon TE300 wide-field microscope equipped with a digital color CCD camera. (D) The effect of FasL and FADD expression on primary human glioma cells was examined. The percentage of cell death in primary glioma cells infected with the various amplicon viral vectors was determined after 72 h. Data shown are averages of triplicate experiment + SEM.

Since many of the GBM-derived cell lines have been propagated in the laboratory for an extensive period of time, we further challenged the clinical application of our viral vectors by testing their functional abilities in primary glioma cells that were isolated directly from the operating theatre. By doing so, we excluded any possible artifact originating from long-term culture *in vitro*, and provided a better evaluation of our viral vectors in a setting that closely resembled the clinical samples in term of preserving the heterogeneous characteristics of glioma cell phenotypes. These primary cultures of human patient-derived glioma cells exhibited constant proliferation rates for a few passages *in vitro *as shown by the positive immunoreactivity with the cellular proliferation marker, Ki67 (Figure 2A, top panel). They also retained the glial cell-specific marker, GFAP (Figure 2A; middle panel), and can be easily infected by the amplicon viral vectors (MOI of 0.8) as shown by the high percentage of enhanced green fluorescent protein (eGFP) positive cells observed (~ 77 %; Figure 2A). FasL expression in the primary human glioma cells resulted in approximately 30 % cell death (Figure 2B) in pG8-*FasL*-infected cells in comparison to those infected with the pG8-18 amplicon vector, which was further confirmed in the TUNEL staining whereby only pG8-*FasL*-infected cells (eGFP+) were TUNEL positive (Figure 2C). By contrast, pG8-18 infected cells exhibited minimal cell death (Figure 2B and 2C). Taken together, we have clearly demonstrated that the pG8-*FasL *amplicon viral vectors induced apoptosis not only in GBM-derived cell lines but also in proliferating primary human glioma cells.

Next, we investigated whether the therapeutic efficacy mediated by pG8-*FasL *could be enhanced by another pro-apoptotic gene such as *FADD *since the Fas/FasL receptor pathway converges at FADD. As shown in Figure 2D, the co-expression of FasL and FADD resulted in the highest level of cell death observed. Cell death induced by pG8-*FasL *and pG8-*FADD *was higher than those observed in pG8-18 infected cells by approximately 3.1-fold and 1.8-fold respectively. The co-expression of FasL and FADD further increased apoptosis by 2.3-fold and 1-fold relative to pG8-*FADD *or pG8-*FasL *alone, respectively. Taken together, these results showed that the co-expression of FasL and FADD synergistically enhanced apoptosis in primary human glioblastoma cells.

### Co-expression of FasL and FADD in vivo prolonged the survival of orthotopic glioma-bearing mice

**Figure 3:**
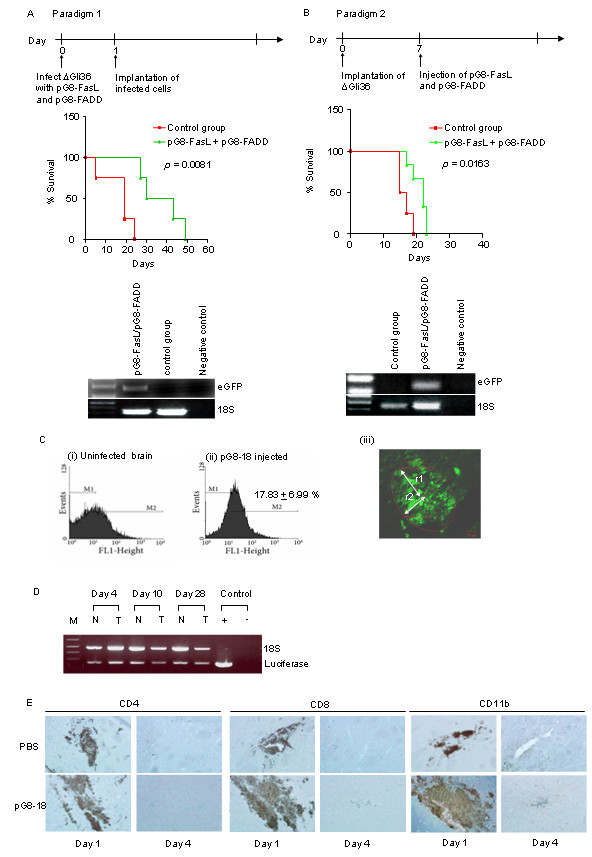
**Effect of FasL and FADD expression in human glioma xenograft *in vivo***. Kaplan-Meier survival analysis of mice receiving (A) ΔGli36 cells preinfected with pG8-*FasL *and pG8-*FADD*, (B) intracranial injections of pG8-*FasL *and pG8-*FADD *into pre-established ΔGli36 tumor in comparison to controls (n = 5). PCR analysis performed on Hirt's DNA isolated from the mouse brain using primers against the exogenous *eGFP *gene and normalized using 18S. (C) Flow cytometry analysis of the percentage of eGFP+ cells in mice injected with 1 × 10^6 ^TU of (ii) pG8-18 amplicon vectors in comparison to (i) control. (iii), Photomicrograph of brain section showed the spreading of the pG8-18 viral vector. Image was captured using a 20×/N.A 0.50 Plan Fluor lens mounted on an Axioimager inverted microscope. *r1 *and *r2 *are the distance from the center of the eGFP+ region. The area of dispersion was calculated using the LSM Image browser (Zeiss) based on the formula for ellipse *area = *π*r1*r2. (D) PCR analysis performed on Hirt's DNA isolated from the non-tumor-bearing hemisphere (N) and the tumor-bearing hemisphere (T) of a mouse brain injected with pG8-18 amplicon viral vectors. PCR was performed using primers specific for *luciferase *and *18S*. (E) Immunohistochemistry images showing the immunogenicity of pG8-18 amplicon viral vectors (1×10^6 ^TU) inoculated into immunocompetent Balb/c mice. Sections were stained for CD4, CD8 and CD11b on days 1 and 4 post-injection and counterstained with methyl green. Images shown are original magnification ×200.

Based on the results above, we decided to investigate whether the synergistic effect of FasL and FADD in enhancing apoptosis could be recapitulated *in vivo*. In Paradigm 1, ΔGli36 human glioma cells were pre-infected with equal ratios (5×10^5 ^TU each) of pG8-*FasL *and pG8-*FADD *amplicon viral vectors followed by implantation into the right hemisphere of immunodeficient mice on the next day (Figure 3A). The viability of ΔGli36 cells were confirmed by both trypan blue as well as TUNEL assays (Additional file 2) prior to intracranial implantation. In Paradigm 2, equal ratios (5×10^5 ^TU each) of pG8-*FasL *and pG8-*FADD *amplicon viral vectors were injected into pre-established ΔGli36 tumors one week after tumor implantation (Figure 3B). As expected, co-expression of both FasL and FADD prolonged the median survival time of ΔGli36 tumor-bearing mice. For Paradigm 1, the median survival time of mice were improved by 57 % from 19 days (control group) to 30 days, with 2 mice surviving past 30 days but eventually succumbed at days 43 and 49 (p = 0.0081 by log-rank; Figure 3A). By contrast, direct intratumoral injection of pG8-*FasL *and pG8-*FADD *into pre-established glioma resulted in a reduced but significant overall survival of 37.5% (p = 0.0163 by log-rank; Figure 3B). PCR analysis confirmed the presence of the amplicon virions based on the *eGFP *marker gene. To understand if the difference observed between the two paradigms may be due to possible differences in the transduction efficiency, the percentage of viral vectors infected cells (as marked by the presence of eGFP) were examined in both scenarios. In Paradigm 1, approximately 95 % of ΔGli36 cells were efficiently infected at an MOI of 2.0 *in vitro *(Additional file 2). By contrast, direct inoculation of the viral vectors resulted in approximately 17.83 % of the glioma cells positive for eGFP (Figure 3Cii). The observed difference may be due to the poor spreading of these vectors or that the vectors were inoculated into region of necrosis or hypoxia that is unfavorable for infection. Thus, we first determined the extent of the viral spread by estimating the area covered by eGFP+ cells in representative cryosections. The estimated area of spread, based on the formula π*r1*r2, (where r1 and r2 represent radii of the eGFP section; Figure 3Ciii), was approximately 0.53 mm^2^, which was less than 0.5% of an average coronal mouse brain section, indicating that the limited vector spread resulted in a lower transduction efficiency. Next, we examined whether these virions were less stable in the tumor-bearing region of the mouse brain. To address this issue, similar amount of viral vectors (1 × 10^6 ^TU) was administered into the normal and glioma-bearing hemisphere of the mouse brain. At different time points, the brains were removed and analyzed for the presence of the *luciferase *gene. As shown in Figure 3D, *luciferase *gene was detectable in both hemispheres up to day 28, indicating the stability of the amplicon viral vectors *in vivo*. We did not check for the presence of the *luciferase *gene over longer time point because mice bearing the ΔGli36 glioma xenograft succumb to brain tumors after 1 month. Although HSV-1 amplicon viral vectors have been reported in several studies to exhibit minimal cytotoxicity due to the absence of the helper viruses, the presence of exogenous elements such as the yeast Gal4 protein and the mouse NF-YA proteins from the pG8-based amplicon viral vectors could potentially generate antigenic peptides that elicit an immune response from the residual nonspecific immune system of the nude mice, which may subsequently also affect the therapeutic outcome in Paradigm 2. To exclude this possibility, pG8-18 amplicon viral vectors (1× 10^6 ^TU) were intracranially administered into immunocompetent Balb/C mice. In parallel, similar volume of PBS was injected into the same region of the mouse brain in the control mice. All mice were sacrificed either on day 1 or 4 post-viral transduction. The activation of the immune response was determined by immunohistochemistry staining for T lymphocytes (CD4 and CD8) and microglia (CD11b) infiltration. CD4, CD8 and CD11b expression were detectable one day post-injection (Figure 3E) in both PBS and pG8-18-injected mice, indicating that both PBS and pG8-18 viral vectors induced inflammatory responses, possibly due to the transient disruption of the blood brain barrier. However, immunoreactivity from the three markers was not detectable at day 4 in neither group of mice, suggesting that pG8-based viral vectors are also relatively non-immunogenic. Taken together, these results demonstrated that the pG8-based viral vector is relatively stable and non-immunogenic *in vivo *and that the lower therapeutic efficacy observed in Paradigm 2 is due to limited vector spread and mode of vector delivery.

### pG8-18 amplicon vector remains functional after treatment with Temozolomide or γ-irradiation

**Figure 4:**
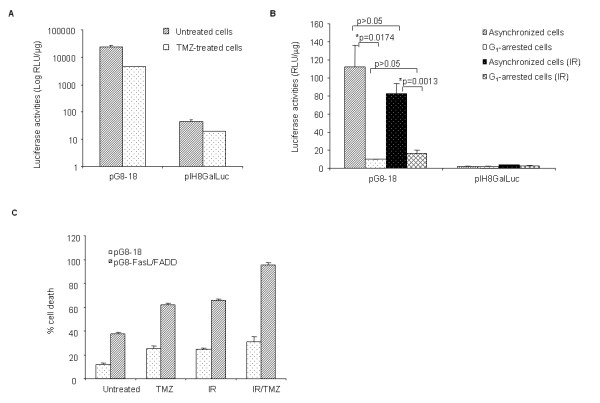
**Effect of TMZ and IR on gene expression mediated by pG8-18-based vectors**. (A) ΔGli36 cells were infected with either pG8-18 or pIH8Gal*Luc *vector for 6 h prior to treatment with TMZ. Cells were treated with TMZ for 1 h, after which the cells were replenished with fresh medium without TMZ and harvested at 48 h post-TMZ treatment for the analysis of luciferase expression. (B) To determine the effect of IR on the cell cycle-regulated transgene expression, ΔGli36 cells were transfected with either pG8-18 or pIH8Gal*Luc *vector for 6 h prior to 3Gy of irradiation, after which the cells were replenished with either fresh medium or medium containing lovastatin. Cells were harvested at 48 h post-transfection for the analysis of luciferase expression. (C) ΔGli36 cells infected with pG8-*FasL *and pG8-*FADD *were subjected to 75 μM TMZ, 3 Gy IR, or combination of TMZ and IR. Percentage of cell death was determined after 72 h. Data shown are % cell death in either pG8-18-infected or pG8-*FasL*/*FADD-*infected cells treated with TMZ, IR or TMZ and IR. For all experiments, data shown are averages of triplicate experiments + SEM and the total amplicon viruses used was MOI of 1.0.

Temozolomide (TMZ) is a monofunctional alkylating agent with a favorable toxicity that is commonly used in the treatment of recurrent glioma [24]. Ionizing radiation (IR) therapy has also been adopted as a choice of treatment for malignant gliomas [25]. Together, they conferred survival benefit in patients with GBM and have become part of a new standard of care for GBM patients [26]. Given that our viral vectors (pG8-18-based vectors) mediated therapeutic gene expression specifically in proliferating glial cells, they are best suited as an adjuvant therapy to kill residual dividing glioma cells that have not been completely removed during surgery, radiation or chemotherapy. Thus, we extended our previous studies to examine the functionality of pG8-18 amplicon vectors in the presence of either treatment. Human glioma ΔGli36 cells were infected with either pG8-18 or the control amplicon viral vector pIH8Gal*Luc *followed by treatment with TMZ. In agreement with published literature [27], we observed an accumulation of cells in the G_2_/M phase (from 24-26 % to 45-51 %) of the cell cycle when the cells were treated with TMZ (data not shown). Although we observed a decline in the luciferase reporter activities in pG8-18-transduced TMZ-treated cells when compared with the pG8-18-transduced untreated cells, similar trend was also observed in cells infected with control pIH8Gal*Luc *vectors (Figure 4A), suggesting that the lower luciferase activity is probably due to the general cytotoxic effect seen in most chemotherapy drugs. Importantly, the overall luciferase activities mediated by pG8-18 is still significantly higher (511-fold) than TMZ-treated glioma cells transduced by the control pIH8Gal*Luc *vectors (Figure 4A). Based on these observations, we concluded that the amplicon viral vectors are functional in the presence of TMZ.

Next, we examined whether DNA damaging γ-irradiation will lead to the loss of previously characterized cell cycle-dependent transgene activation conferred by pG8-18 vectors [4]. To minimize potential virus-specific interference, we have chosen to transfect the ΔGli36 human glioma cells with pG8-18 amplicon vectors, followed by 3 Gy of γ-irradiation. As shown in Figure 3B, significant differences in luciferase activities were not observed between the untreated and the irradiated cells transfected with pG8-18, both in the asynchronized and in the G_1_-arrested populations. Furthermore, IR did not severely hamper the cell cycle-regulated luciferase gene expression mediated by pG8-18; the luciferase expression was significantly higher in the IR-treated asynchronized cells (82.45 RLU/μg) compared with IR-treated G1-arrested cells (16.28 RLU/μg) (Figure 4B). Our results showed that γ-irradiation did not negatively affect the cell cycle-regulated luciferase expression mediated by the pG8-18 vector. Taken together, these results confirmed that pG8-18-based amplicon vector could be used in combination therapies involving TMZ/IR or both.

To determine whether concurrent treatment of the therapeutic vectors, pG8-*FasL *and pG8-*FADD*, with TMZ or IR or both could induce an even greater extent of cell death, ΔGli36 glioma cells were pre-infected with MOI of 1.0 of pG8-*FasL *and pG8-*FADD *amplicon viruses, followed by treatment with TMZ, IR or both. In parallel, pG8-18-infected cells were treated the same way and used as controls. Our results showed that the percentage of cell death induced by the pG8-18 with TMZ (24.8 %) or IR (24.7 %) alone was lower in comparison to pG8-*FasL*/*FADD *amplicon viruses (37.6 %; Figure 4C). However, when the pG8-*FasL*/*FADD *was introduced together with TMZ and IR, the apoptotic activity of pG8-*FasL*/*FADD *was significantly enhanced from 37.6 % (untreated) to 62 % (TMZ), 66 % (IR) and 95.5 % (TMZ/IR), respectively (Figure 4C). These results showed that TMZ and IR further increased the efficacy of *FasL *and *FADD in vitro*.

### Double arm therapy using pG8-FasL/FADD with TMZ prolonged the survival of glioma-bearing mice

**Figure 5:**
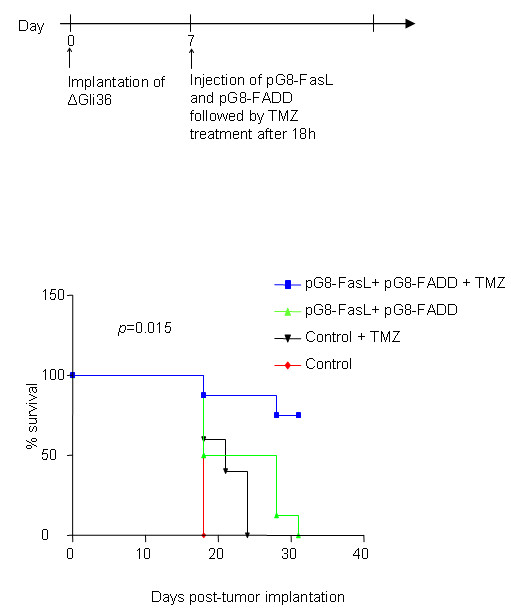
**Effect of TMZ on FasL and FADD-mediated tumor regression in intracranial glioma model**. Kaplan-Meier survival analysis of mice receiving ΔGli36 human glioma cells treated with pG8-*FasL *and pG8-*FADD *in combination with TMZ. Animals were treated on day 7 post-tumor implantation with 2×10^5 ^TU (MOI 1.0) of either pG8-*FasL *and pG8-*FADD *or pG8-18 injected i.t. followed by i.p. delivery of TMZ (5 mg/kg bodyweight) after 18 h. N = 8. Log rank test, *p < 0.05*.

To confirm the observed enhanced efficacy of pG8-*FasL *and pG8-*FADD *in the presence of TMZ and IR *in vivo*, the amplicon viruses were administered one week post-tumor cells implantation subcutaneously. The follow day, TMZ was given for a period of 5 days at 10 mg/kg/day, and IR was administered at 2 Gy/day for the same period of time (Additional file 3). Our results showed that the pG8-*FasL *and pG8-*FADD *amplicon viruses could effectively suppressed the tumor growth for about 1 week post-injection. However, the anti-tumor effect was lost as the tumor grew in size, which was anticipated since these amplicon viruses are defective in replication. Mice treated with both amplicon viruses and IR resulted in tumors about 1/4 the size of control alone. Complete tumor regression was observed in animals injected with pG8-*FasL/FADD *followed by combined treatment with TMZ and IR. So far, the data are in agreement with our earlier *in vitro *findings. Contrary to our expectation, the group of mice treated with pG8-*FasL/FADD *and TMZ also had tumors that regressed completely. We attributed the latter to the high dose of TMZ used that had efficiently masked the precise therapeutic effect of the amplicon viruses. Since post-treatment with IR exhibited minimal effectiveness in this experiment, we decided to focus our study on evaluating the efficacy of amplicon viruses with TMZ treatment in a clinical relevant mouse model. Accordingly, pG8-*FasL/FADD *amplicon viruses were intracranially administered to mice bearing intracranial high grade gliomas as depicted in Figure 5. TMZ (5 daily doses of 5 mg/kg) was administered 8 days post-tumor implantation. As shown in Figure 5, combination treatment of TMZ with pG8-*FasL/FADD *significantly prolonged the survival of the tumor-bearing mice in comparison to TMZ or pG8-*FasL/FADD *alone. On the other hand, TMZ, as a single agent, was not as effective as pG8-*FasL/FADD *in enhancing the survival of tumor bearing mice, albeit the difference between the median survival time for both treatment regimens was not statistically significant. Collectively, our results showed that TMZ can be used in combination with the cell cycle-regulated vector to markedly improve anti-tumor response.

## Discussion

GBM have retained their dismal prognosis despite advances in neurosurgical techniques, radiation and drug therapies. Some of the difficulties encountered include inaccessibility to resective surgery because of anatomical location and tumor recurrences. Based on a model that predict the number of tumor cells distributed around the primary tumor bed, the percentage of tumor cells found at a distance more than 2 cm from the tumor edge is at ~2 % prior to surgery and increased to ~ 23 % post-surgical resection [28]. Thus, a strategy that can effectively target the highly proliferating tumor cells is urgently in need. We have previously generated a HSV-1 amplicon viral vectors whereby the transgene expression is regulated by cell proliferation [4,6]. The present study aimed to explore the clinical feasibility of this vector in the treatment of human brain tumors by placing the *FasL *or *FADD *genes under the regulation of a glial cell-specific promoter. We demonstrated the therapeutic efficacy of these vectors in primary cultures of human brain tumors, and showed their ability to mediate cell-type specific transgene expression *in vivo*. More importantly, the efficacy of these therapeutic viruses was greatly enhanced by TMZ, resulting in prolonged survival of glioma-bearing mice.

The ligands of the TNF family (e.g, FasL, TRAIL and TNF-α) and the members of the corresponding TNF receptor superfamily are known to exhibit pleiotrophic activities in mammalian cells. They can induce cellular proliferation, differentiation or cell death depending on the responding cell type and the microenvironment [7], for e.g., Fas/FasL interaction has been shown to be involved in neurogenesis [29]; the Fas/FasL system was also demonstrated to confer immune privileged status to tumor cells due to the expression of FasL on the tumor cells and the tumor endothelium [30,31], which induces cell death in the Fas-expressing T cells. However, how this process is regulated is still unknown. Despite these issues, several groups have generated recombinant viral vectors that deliver the *FasL *gene to eradicate glioma cells and have demonstrated prolongation of survival [32,33]. However, high level of FasL expression has been shown to induce liver failure [34]. Thus, restricting the FasL expression to tumor cells is essential if FasL is to be employed for cancer therapy. We have chosen *FasL *as our therapeutic gene because in our cell cycle-dependent transgene activation system, the therapeutic effect cannot be overwhelming as this could potentially mask the cell cycle-regulatory property of the vectors. To circumvent the possible complications of the immune system, we have chosen the immunodeficient nude mice as our mouse model; hence, the *FasL*-induced apoptosis could still serve as a good tool to assess the therapeutic efficacy of our dual-function viral vectors.

Many clinical trials in human brain tumors are conducted by injecting recombinant viral vectors into the tumor cavity margins following surgical resection [35]. It is therefore important that (i) the tumor cells are susceptible to viral infection; (ii) the viruses are stable without causing adverse cytotoxic effects; and (iii) the transgene expression is restricted to only tumor cells. We have demonstrated that transduction efficiency of pG8-18 amplicon viruses was relatively high in proliferating GFAP-positive primary human glioma cells. However, the transduction efficiency of HSV-1 amplicon viral vectors has been reported to vary in different primary glioma cell cultures, possibly due to the heterogeneity of the glioma cells and the variation in the cell surface receptors required for viral entry [36]. Thus, it may be necessary to pre-examine the efficiency of infection on a patient's tumor sample. We have also demonstrated that the amplicon viruses are relatively stable (Figure 3D) although the transgene expression mediated by these vectors maybe transient due to the increasing tumor cells to vector ratio. The *GFAP *enhancer sequence has been shown to confer glial-cell specificity to T98 [37], ΔGli36, U251 and SF767 [4]. Because the *GFAP *enhancer elements drive FasL and FADD expression specifically in glial cells, the packaging efficiency of the virions was unaffected (data not shown). Furthermore, FasL expression mediated by pG8-*FasL *vectors was higher in proliferating versus growth arrested ΔGli36 human glioma cells (Figure 2B), which correlated with the enhanced apoptosis observed in the proliferating GFAP-positive ΔGli36 cells (Figure 2A). By contrast, FasL expression did not differ in the proliferating HeLa cells versus the G_1_-arrested cells (Figure 1D), indicating that the transgene expression mediated by pG8-*FasL *is regulated by type of cells under proliferating conditions. This is further supported by similar finding *in vivo *(Figure 1E).

One of the major obstacles encountered in targeting death receptors in tumor cells is that the cells are usually resistant to apoptosis induced by death receptor ligands [17,38]. A recent report has shown that CD133-positive cells isolated from human glioma cells are also resistant to *Fas*-induced apoptosis [39]. Likewise, cells derived from human patients have been shown to be resistant to etoposide, paclitaxel, TMZ and carboplatin [40]. These findings suggest that the immature stem cells in glioma could be an important factor of resistance to *Fas *signaling pathway. Thus, enhanced therapeutic efficacy is much desired. The co-expression of FADD and caspase-8 are reported to be required for the synergistic cytotoxicity induced by combined IR/TRAIL treatment [41]. As such, we explored whether the therapeutic efficacy of pG8-*FasL *could be improved in the presence of *FADD*. Our results showed that the co-expression of FasL and FADD in primary glioma cells enhanced apoptosis by 20% *in vitro *(Figure 2D) and prolonged the survival of intracranial glioma bearing mice (Figure 3A and 3B). However, the therapeutic efficacy varies depending on whether the viruses were used to infect human glioma cells prior to tumor cell implantation (Paradigm 1) or after the establishment of the tumor mass (Paradigm 2). In both scenarios, significantly prolonged survival was observed in mice treated with amplicon viruses albeit paradigm2 was substantially less effective. The latter was attributed to the limited vector spread and mode of vector delivery but not due to the instability of the amplicon virions (Figure 3A, B and 3D) or possible immunocytotoxicity elicited by the vectors (Figure 3E). In fact, Suzuki et al. has reported the persistent transgene expression conferred by HSV-1 amplicon vectors in the brains of immunocompetent C57BL/6 mice (up to 385 days post viral injection) through the incorporation of the Epstein Barr Virus (EBV) episomal elements [42].

TMZ in combination with IR are currently the first-line treatment for recurrent GBM and when used concurrently, have been shown to improve the median survival time of glioma patients for up to 5 years of follow-up [43]. With that in mind, we investigated whether TMZ and IR can improve the overall cell death induced by pG8-*FasL *and pG8-*FADD *amplicon viruses. Indeed, combination treatment of ΔGli36 cells with pG8-*FasL/FADD *amplicon viral vectors, TMZ and IR markedly enhanced the percentage of cell death by ~ 40 % (Figure 4C). We further challenged the effectiveness of TMZ and IR *in vivo *(Additional file 3). This time, the suppressive effect of the therapeutic amplicon viruses was not as remarkable as shown previously (Figure 1E), possibly due to the lower dose of viruses used, a different derivative of glioma cells used and a different strain of mice (SCID mice versus nude mice). Despite these variable parameters, pG8-*FasL/FADD *amplicon viruses can still mediate a suppressive effect on the tumor growth. Irradiation, however, did not significantly enhance the overall therapeutic efficacy mediated by the pG8-*FasL/FADD *viruses (Additional file 3). This is similar to a report by Yamini et al in that IR alone with adenovirus-delivered tumor necrosis factor (TNF) did not improve the survival of glioma-bearing mice [44]. Since the concomitant and adjuvant dosage of TMZ and IR with pG8-*FasL*/*FADD *is difficult to manipulate *in vivo*, we decide to focus our study on the effect of TMZ and pG8-*FasL*/*FADD *in mice bearing intracranial gliomas, which are of more clinical relevance. Our results showed that adjuvant TMZ boosted the therapeutic efficacy of pG8-*FasL*/*FADD*; the survival time was markedly prolonged in comparison to mice receiving either TMZ or pG8-*FasL*/*FADD *viruses alone (Figure 5).

The effectiveness of TMZ is largely determined by the status and expression level of the O^6^-methylguanine-DNA methyltransferase (MGMT) [45]. Silencing of the *MGMT *promoter has been shown to confer therapeutic benefits by inhibiting DNA repair upon DNA damage induced by TMZ [46]. Moreover, Hegi et al showed that the patients with methylated *MGMT *promoter has better survival than those without after TMZ and IR treatment [47]. Therefore, we speculated that an even greater therapeutic efficacy of pG8-*FasL/FADD *and TMZ could be achieved in human glioma cells with generally low MGMT activity. Alternatively, a greater effect may be seen by increasing the viral dosages, or using a more potent therapeutic gene such as the *caspase-8 *or bacterial exotoxin. Caspase-8 is frequently lost or silenced in human gliomas [20]. Inducible caspase-8 has been shown to be effective in prostate cancer gene therapy [48] and malignant brain tumors [49]. A fusion protein consisting of interleukin 13 (IL-13) and a mutated form of *Pseudomonas *exotoxin (IL-13-PE) has also been shown to induce potent and specific cytotoxicity in glioma cells that overexpresses the receptor for IL-13, IL13 receptor-α2 (IL13-Rα2) [50]. Since our cell cycle-regulatable HSV-1 amplicon viral vectors have been shown to confer relatively tight regulation of gene expression, it will be interesting to study the potential efficacy in these settings.

From a clinical application point of view, there are two ways one could use these vectors as gene delivery vehicles. They can either be used directly to infect cells surrounding the margins of tumor resection followed by adjuvant/concurrent treatment with TMZ and/or IR, or to infect *ex-vivo *cultured adult human mesenchymal stem cells (MSC), which has been shown to be resistant to chemotherapy drugs such as cisplatin, vincristine, and etoposide [51] and IR [52]. Although the latter strategy needs to be stringently evaluated, the inherent tumor tracking properties of MSC is extremely attractive especially since the incidence of metastatic brain tumors with high proliferative potential is predicted to increase [53]. We have performed independent studies to show that HSV-1 amplicon viral vectors can infect MSC efficiently without affecting the cellular proliferation, tumor homing and multilineage differentiation potential of MSC [54]. Thus, further studies are required to couple the homing potential of MSC with the cell cycle-regulatable HSV-1 amplicon vectors.

In summary, we have demonstrated the therapeutic efficacies of pG8-*FasL/FADD *amplicon viruses in human glioma cells derived from established cell lines and patients biopsy samples. The vectors are relatively stable with minimal cytotoxicity and remained functional in the presence of chemotherapy and ionizing radiation treatment. More importantly, combined treatments of these therapeutic viruses with TMZ prolonged the survival of intracranial glioma-bearing mice. Given that gliomas are heterogeneous in nature, the combination of TMZ and our cell cycle-regulated *FasL *and *FADD *vector should confer added survival benefits.

## Conclusion

We have previously constructed a HSV-1 amplicon viral vector in which the transgene expression is regulated by cellular proliferation. In the present study, we demonstrated that Ki67-positive proliferating primary human glioma cells cultured from biopsy samples were effectively induced into cell death by the dual-specific function of pG8-*FasL *amplicon vectors. These vectors are cell-type specific in addition to their ability to confer cell cycle-dependent transgene expression. Their efficacies are not hampered by the presence of chemotherapy or irradiation, and are relatively stable and non-cytotoxic *in vivo*. Most importantly, the combined therapies of pG8-*FasL *and pG8-*FADD *in the presence of TMZ significantly improved the survival of mice bearing intracranial high-grade gliomas. In summary, these amplicon viral vectors are potentially useful as adjuvant therapy to complement the current therapeutic regimens for human gliomas.

## Materials and methods

### Isolation of primary human glioma cells

This study has been approved by the SingHealth Centralized Institutional Review Board, Singapore. Primary human glioma cells were isolated, after informed consent, from the brain tumor tissues of patients undergoing brain tumor surgery at the National Neuroscience Institute, Singapore. The harvested tissue was separated into small pieces in the presence of complete medium (Astrocyte Basal Medium (ABM) supplemented with 10% FBS, Penicillin/Streptomycin, normocin and L-Glucose; Cambrex Bio Science Walkersville, Inc., Walkersville, MD). The tissue suspensions were first passed through a 5 ml serological pipette, followed by a 1 ml pipette and finally a flame-polished pasteur pipette until no clumps were visible. Following trypsin digestion, the homogenate was filtered through a 70-μm cell strainer (BD Biosciences, San Jose CA), and then subjected to centrifugation. The collected cells were cultured in complete ABM. All cells were maintained at 37°C in a humidified incubator with 5% CO_2_. The culture of ΔGli36 and HeLa cells was performed as described previously [6].

### Plasmid constructions

The construction of pG8-18, pIH8Gal*Luc*, pC8-36, pC8-*FasL*, and pIH8Gal*FasL *plasmid were described previously [4,6]. To generate pG8-*FasL*, the entire DNA fragment encoding the *Gal4/NF-YA *fusion protein and the 8Gal*FasL *region from pC8-*FasL *vector [6] was excised using *Pme*I and inserted into the same restriction enzyme site on pG8-18. A similar subcloning strategy was used for the construction of pG8-*FADD *from pC8-*FADD *[6]. All plasmids were amplified in *E. coli *STBL-2 (Invitrogen, Grand Island, NY) and the DNA was extracted using a QIAprep Spin Miniprep kit (Qiagen GmbH, Hilden, Germany) and verified by DNA sequencing (Applied Biosystem Inc., USA).

### Synchronization of cells for cell cycle analysis

Synchronization of cells in the G_1 _phase of the cell cycle was performed by treating the cells with 40-60 μM of lovastatin (Merck, Singapore) in the presence of 0.1% FBS for 48 h. Cell cycle analysis was performed as described previously [4].

### Packaging of helper virus free HSV-1 amplicon viral vectors

Packaging of the HSV-1 amplicon vector was performed as described previously using the helper virus-free packaging method [55]. The titer obtained for the resulting packaged amplicon viral vectors ranged from 1×10^7 ^to 1×10^8 ^TU/ml after concentration through a sucrose gradient. Infection of viral vectors on ΔGli36 and HeLa cells were performed at a multiplicity of infection (MOI) of 1.0 and the transduction efficiency was determined by flow cytometry for the presence of eGFP+ cells.

### Immunohistochemistry, immunofluorescence and TUNEL staining

Immunohistochemistry and immunofluorescence staining were performed as previously described [4]. Antibodies (GFAP, Ki67, CD4, CD8, and CD11b) were purchased from BD Biosciences and used at 2 μg/ml concentration. TUNEL staining was performed using the In situ cell death detection kit (Roche) according to manufacturer's instruction. Briefly, fixed cells were permeabilized with 0.1% triton in 0.1% sodium citrate solution prior to incubating in solution containing the terminal deoxynucleotidyl transferase enzyme and nucleotide mixture. Staining of cells was carried out at 37°C for 1 h. After which, non-specific staining was removed by rinsing the cells in PBS twice. Samples were then visualized under fluorescence microscope. All images were either acquired on the CCD digital camera (Olympus DP11, Olympus, Japan) mounted on the upright microscope (Olympus BX41) or the Nikon TE300 Eclipse fluorescence microscope (Nikon, Japan).

### Treatment with TMZ and γ-irradiation

Temozolomide (Temodal; Schering Plough, Belgium) was dissolved in DMSO (Sigma Aldrich) to produce a 100 mM stock solution for *in vitro *experiments. For *in vitro *experiments, TMZ was diluted with PBS to obtaine 75 μM solutions. For *in vivo *experiments, stock solution was diluted in PBS to a final concentration of 5 mg/ml. A dose of 5 mg/kg body weight was used, which is equivalent to half of the recommended dosage of 25 mg/kg/m^2 ^in adult humans [56].

To assess the effect of TMZ treatment on *luciferase *gene expression, cells were first infected with the respective amplicon viral vectors for 6 h. The transduced cells were divided into two groups, one portion of the cells were cultured in complete medium, while the other portion was treated with 75 μM of TMZ at 37°C. After 1 h of treatment, the cells were rinsed twice with PBS and replenished with complete medium containing 10% serum. To assess the effect of IR, similar procedure was performed as treatment with TMZ, except that the cells were transfected with pG8-18 or pIH8Gal*Luc *plasmid. Transfected cells were exposed to 3 Gy of γ-irradiation, followed by incubating the cells in either fresh complete medium or medium containing lovastatin. Luciferase activities were measured after 48 h of transfection.

To assess the effect of TMZ and IR on pG8-*FasL/FADD*-mediated cell death, ΔGli36 cells was infected with MOI of 1.0 of pG8-*FasL *and pG8-*FADD *or pG8-18 amplicon viruses. The viral supernatant was removed after 6 h and cells were cultured in medium containing 75 μM of TMZ. The cells were subsequently subjected to 3Gy of IR treatment. TMZ-containing medium was removed after 1 h of incubation. The percentage of cell death was assessed after 72 h by trypan blue exclusion assay.

### Animal Experiments

All animal experiments were performed according to the guidelines and protocols approved by the SingHealth Institutional Animal Care and Use Committee, Singapore.

To determine the efficacy of FasL *in vivo*, 6-8 weeks old CB-17 SCID mice (Animal Resource Centre, Australia), inoculated with either HeLa or ΔGli36-SCID8 cells (2 × 10^6^) at their right flank, were divided into 4 groups. One day following tumor inoculation, 2 × 10^6 ^TU of viral vector was administered intratumorally. Injections of viral vectors were repeated every 10 days until tumor necrosis was observed in the non-treated groups. The tumor volume was measured and calculated according to the formula *volume = 0.52 × length × width^2^*. At the end of the experimental period, all animals were sacrificed and tumor nodules were harvested. Analysis of FasL expression was also performed as described previously [6].

To determine the synergistic effect of *FasL *and *FADD *in intracranial tumor-bearing mice, two experimental paradigms were designed. In Paradigm 1, ΔGli36 human glioma cells (1×10^6^) were pre-infected with equal ratios (5×10^5 ^TU each) of pG8-*FasL *and pG8-*FADD *amplicon viral vectors followed by implantation into the right hemisphere (bregma (0,0) lateral 2.0 mm and depth 2.5 mm) of immunodeficient nude mice on the next day. For Paradigm 2, mice were inoculated with ΔGli36 intracranially followed by inoculation of amplicon viral vectors (MOI of 1.0) intratumorally 7 days later. Mice were monitored weekly for changes in body weight.

For investigating the combined therapeutic effect of TMZ with pG8-*FasL *and pG8-*FADD in vivo*, immuno-incompetent nude mice were first inoculated with 2×10^5 ^ΔGli36 cells intracranially. Amplicon viruses, either pG8-18 (2×10^5 ^TU) or pG8-*FasL *and pG8-*FADD *(MOI of 1.0, 1×10^5 ^TU each), were injected intratumorally (i.t.) into the same co-ordinates after 1 week. TMZ was administered after 18 h and on a daily basis for 5 days (5 mg/kg which total to 25 mg/kg) via an intraperitoneal (i.p.) route.

To assess the effect of TMZ and/or IR on FasL and FADD-mediated tumor regression in a subcutaneous glioma model, mice bearing ΔGli36 human glioma xenograft (5×10^5^) at their hind limbs were randomized into groups indicated in Additional File 3. One week post tumor cell implantation, pG8-*FasL *and pG8-*FADD *amplicon viruses (combined MOI = 1) were injected intratumorally (i.t). Treatment with TMZ, IR or TMZ and IR was initiated 18 h post-virus inoculation. TMZ was delivered i.p. at a dose of 10 mg/kg/day for 5 doses, and IR (2Gy/day) was given to the mice, which amount to a total of 10 Gy. Tumor volume was measured every 3-4 days as detailed above.

To determine the stability of the pG8-18 amplicon viral vectors *in vivo*, 1×10^6 ^TU of pG8-18 vector was injected into the left (control) and right hemisphere of the same immunodeficient nude mice. The right hemisphere consisted of tumor mass (1 × 10^6 ^ΔGli36), which was implanted a week before viral injection. The brains were then harvested on days 4, 10, and 28 post-injection and prepared for extraction of viral DNA. Viral DNA was recovered from brain tissues using Hirt's method [57] with slight modifications. Briefly, the tissues were first frozen with liquid nitrogen and ground into powder using a mortar and pestle. After that, the tissues were incubated in 500 μL of lysis buffer (0.6 % SDS, 10 mM EDTA, pH 8.0, 10 mM Tris-HCl, pH 7.5) for 20 min at room temperature followed by addition of 125 μl of 5 M NaCl and incubated at 4°C overnight. The next day, the extract was subjected to centrifugation at 13 000 × g at 4°C for 30 min. The supernatant was recovered and extracted with phenol, phenol-chloroform and chloroform. The DNA was precipitated using isopropanol, rinsed with 70% ethanol, and dissolved in 35 μl of TE (10 mM Tris-Cl pH8.0, 1 mM EDTA) buffer.

To determine the transduction efficiency of pG8-18 amplicon viral vectors, immunodeficient nude mice were separated into 2 groups and inoculated with 10 μl of pG8-18 (1 × 10^6 ^TU) viral vector. The mouse brains were harvested 1 day post-injection. On the day of harvesting, the mice were first perfused through the heart with PBS, and the brains were harvested and processed to single cell suspensions. The brains were homogenized in a 50 ml falcon tube with 12 ml of HBSS (Invitrogen) using a 5 ml serological pipette, followed by a 1 ml serological pipette and a flamed-polished Pasteur pipette, until no clumps were visible. Cells were trypsinized and incubated for 15 min at 37°C, with mixing every 5 min. The homogenates were then filtered through a 70-μm pore size nylon cell strainer (BD Biosciences), and the filtrates were subjected to centrifugation at 500 rpm for 15 min at 4°C (without brake) (Beckman Coulter). The supernatant was removed and the cell pellet resuspended in DMEM containing 10% serum. The percentage of eGFP+ cells was analyzed using FACS. For the other group of mice, the brains were fixed in 4 % PFA solution overnight, followed by 30 % sucrose for 48 h, and then sliced into 10-micron sections. The eGFP+ cells were visualized using a LSM 510 Meta confocal microscope (Carl Zeiss Microscopy, Göttingen, Germany) with the appropriate filters.

To determine the immunogenicity of the HSV-1 amplicon viruses, immunocompetent Balb/c mice (6 weeks old) was inoculated with either saline or pG8-18 viruses (1×10^4 ^TU) in the right hemisphere of the mouse brain. Brains were harvested on day 1 and 4 post-injections. On the day of harvesting, mice were perfused through the heart with PBS followed by 4% paraformaldehyde. Brains were processed and cryosectioned at 10-micron thickness. Immunohistochemical staining was performed on consecutive sections.

### Statistical analysis

The data are presented throughout this study as means + standard error of the mean. The statistical significance was evaluated by an unpaired t-test, and p < 0.05 was considered significant. Kaplan-Meier survival analysis was used to calculate the percentage of survival as a function of time, and the survival curves were compared using the log-rank test.

## List of Abbreviations

eGFP: enhanced green fluorescent protein; FADD: Fas associated protein with a death domain; FasL: Fas Ligand; GBM: Glioblastoma Multiforme; GFAP: Glial fibrillary acidic protein; HSV-1: Herpes Simplex Virus type 1; i.p.: intraperitoneal; IR: irradiation; i.t.: intratumoral; TMZ: temozolomide; TU: transduction unit

## Competing interests

The authors declare that they have no competing interests.

## Authors' contributions

IH designed and executed the experiments and took part in writing the manuscript; WHN provided the primary human glioma samples together with relevant clinical information and took part in proofreading the manuscript; PL was involved in the overall design of the experiments, established collaboration, and wrote the manuscript. All authors have read and approved of the final manuscript

## Supplementary Material

Additional file 1 **Schematic diagram of vectors used**. (A) The pC8-36 vector contained the *CMV *promoter driving the *Gal4/NF-YA *transactivator in place of the *GFAP *enhancer element in pG8-18. (B) pC8-*FasL *was generated by removal of the *luciferase *gene from pC8-36 and replaced with the *FasL *gene. (C) The pG8-18 vector contained three-tandem repeats of the *GFAP *enhancer element upstream of the minimal *CMV *promoter. The pG8-18 amplicon vector consisted of the *eGFP *gene under the control of the immediate early promoter (IE4/5p) for titering and monitoring of viral infection. pG8-*FADD *was generated by swapping the *luciferase *transgene in pG8-18 with the *FADD *gene. (D) pIH8Gal*Luc*, which lacked the *Gal4/NF-YA *transactivator sequence, served as a negative control throughout this study. Click here for file

Additional file 2 **Viability and transduction efficiency of ΔGli36 cells prior to implantation**. (A) TUNEL assay was performed on pre-infected cells to confirm the viability of the cells prior to intracranial inoculation. (B) The transduction efficiency of ΔGli36 cells pre-infected with pG8-*FasL *and pG8-*FADD *prior to tumor implantation was determined by FACS analysis. Image shown was pseudocolored. Flow cytometry image of infected cells (red) was superimposed on to the image of uninfected cells (green). Click here for file

Additional file 3 **Effect of TMZ and IR on FasL and FADD-mediated tumor regression**. The effect of TMZ and/or IR on FasL and FADD-mediated tumor regression was examined in a subcutaneous glioma model. Mice bearing ΔGli36 human glioma xenograft (5×10^5^) at their hind limbs were randomized into groups indicated and injected with MOI of 1.0 of pG8-FasL/FADD amplicon viruses (i.t.) one week post-tumor cells implantation. Treatment with TMZ, IR or both was initiated 18 h post-virus inoculation. TMZ was delivered i.p. at a dose of 10 mg/kg for 5 doses, and IR (2Gy) was given to the mice daily to a total of 10 Gy. Tumor volume was measured every 3-4 days. Arrow indicated viral inoculation. Click here for file
