# Supplementary figures and images for: FasL and FADD delivery by a glioma-specific and cell cycle-dependent HSV-1 amplicon virus enhanced apoptosis in primary human brain tumors
Source: Mol Cancer. 2010 Oct 13;9:270. doi: 10.1186/1476-4598-9-270 (PMC2964619; doi:10.1186/1476-4598-9-270)

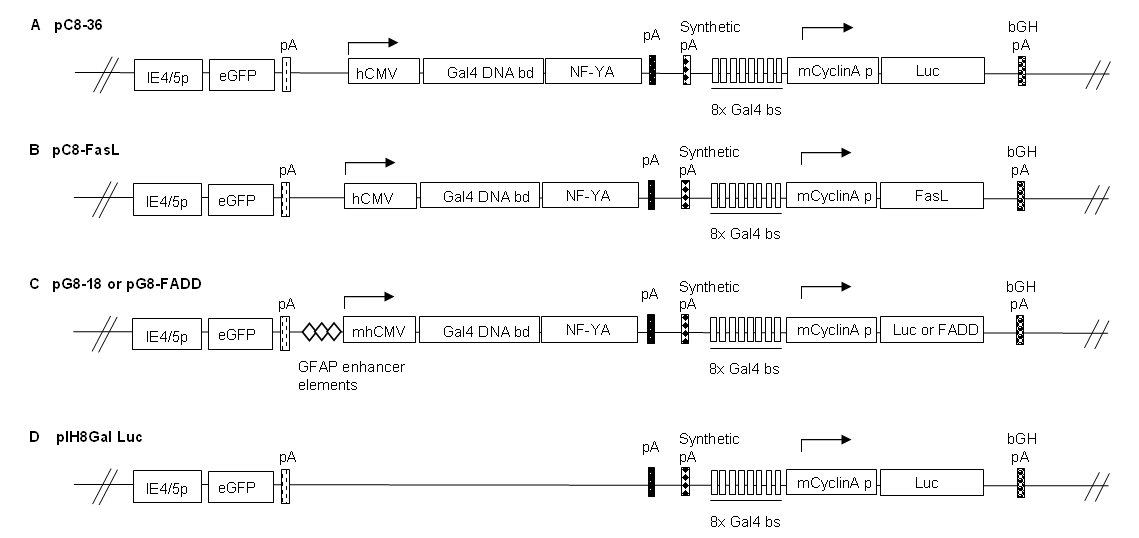

Supplement: Additional file 1 — Schematic diagram of vectors used. (A) The pC8-36 vector contained the CMV promoter driving the Gal4/NF-YA transactivator in place of the GFAP enhancer element in pG8-18. (B) pC8-FasL was generated by removal of the luciferase gene from pC8-36 and replaced with the FasL gene. (C) The pG8-18 vector contained three-tandem repeats of the GFAP enhancer element upstream of the minimal CMV promoter. The pG8-18 amplicon vector consisted of the eGFP gene under the control of the immediate early promoter (IE4/5p) for titering and monitoring of viral infection. pG8-FADD was generated by swapping the luciferase transgene in pG8-18 with the FADD gene. (D) pIH8GalLuc, which lacked the Gal4/NF-YA transactivator sequence, served as a negative control throughout this study. [file 1476-4598-9-270-S1.TIFF]

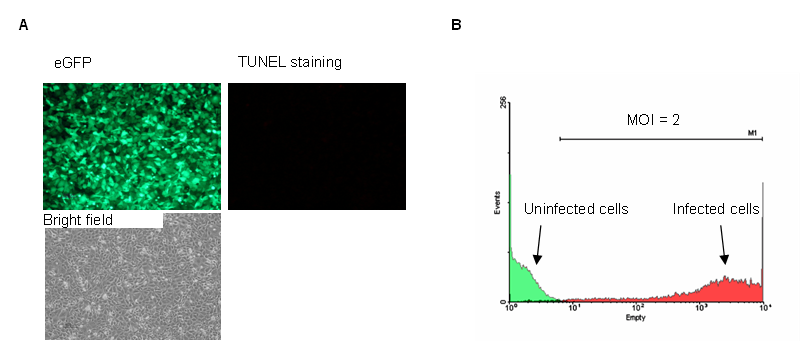

Supplement: Additional file 2 — Viability and transduction efficiency of ΔGli36 cells prior to implantation. (A) TUNEL assay was performed on pre-infected cells to confirm the viability of the cells prior to intracranial inoculation. (B) The transduction efficiency of ΔGli36 cells pre-infected with pG8-FasL and pG8-FADD prior to tumor implantation was determined by FACS analysis. Image shown was pseudocolored. Flow cytometry image of infected cells (red) was superimposed on to the image of uninfected cells (green). [file 1476-4598-9-270-S2.TIFF]

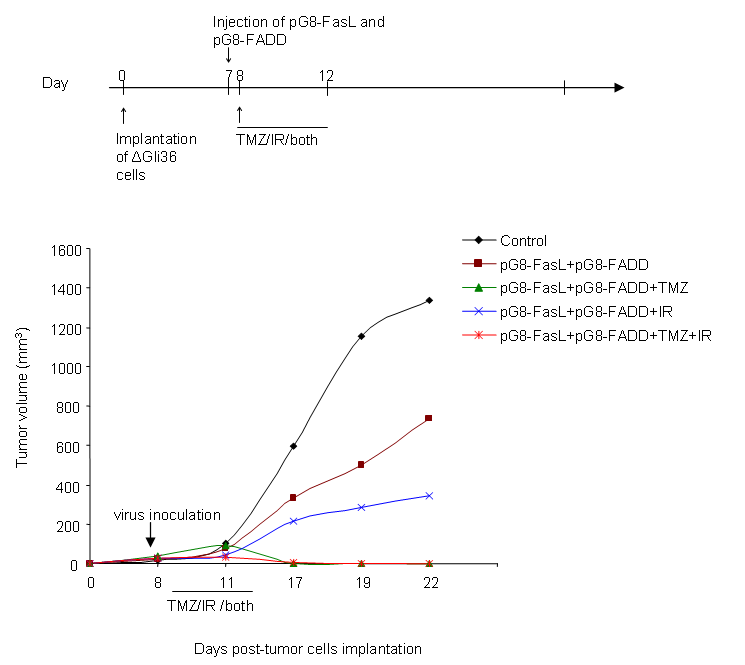

Supplement: Additional file 3 — Effect of TMZ and IR on FasL and FADD-mediated tumor regression. The effect of TMZ and/or IR on FasL and FADD-mediated tumor regression was examined in a subcutaneous glioma model. Mice bearing ΔGli36 human glioma xenograft (5×10^5^) at their hind limbs were randomized into groups indicated and injected with MOI of 1.0 of pG8-FasL/FADD amplicon viruses (i.t.) one week post-tumor cells implantation. Treatment with TMZ, IR or both was initiated 18 h post-virus inoculation. TMZ was delivered i.p. at a dose of 10 mg/kg for 5 doses, and IR (2Gy) was given to the mice daily to a total of 10 Gy. Tumor volume was measured every 3-4 days. Arrow indicated viral inoculation. [file 1476-4598-9-270-S3.TIFF]
